# Supplementary material for: Vitamin D Modified DSS-Induced Colitis in Mice via STING Signaling Pathway
Source: Biology (Basel). 2025 Jun 18;14(6):715. doi: 10.3390/biology14060715 (PMC12190092; doi:10.3390/biology14060715)
Supplement: Supplementary file 1 [file biology-14-00715-s001.zip › Supplementary Materials S1: 16s-report of gut microbiota/Groups/diff_analysis/heatmap/GMMclusterdiffheatmap.pdf]

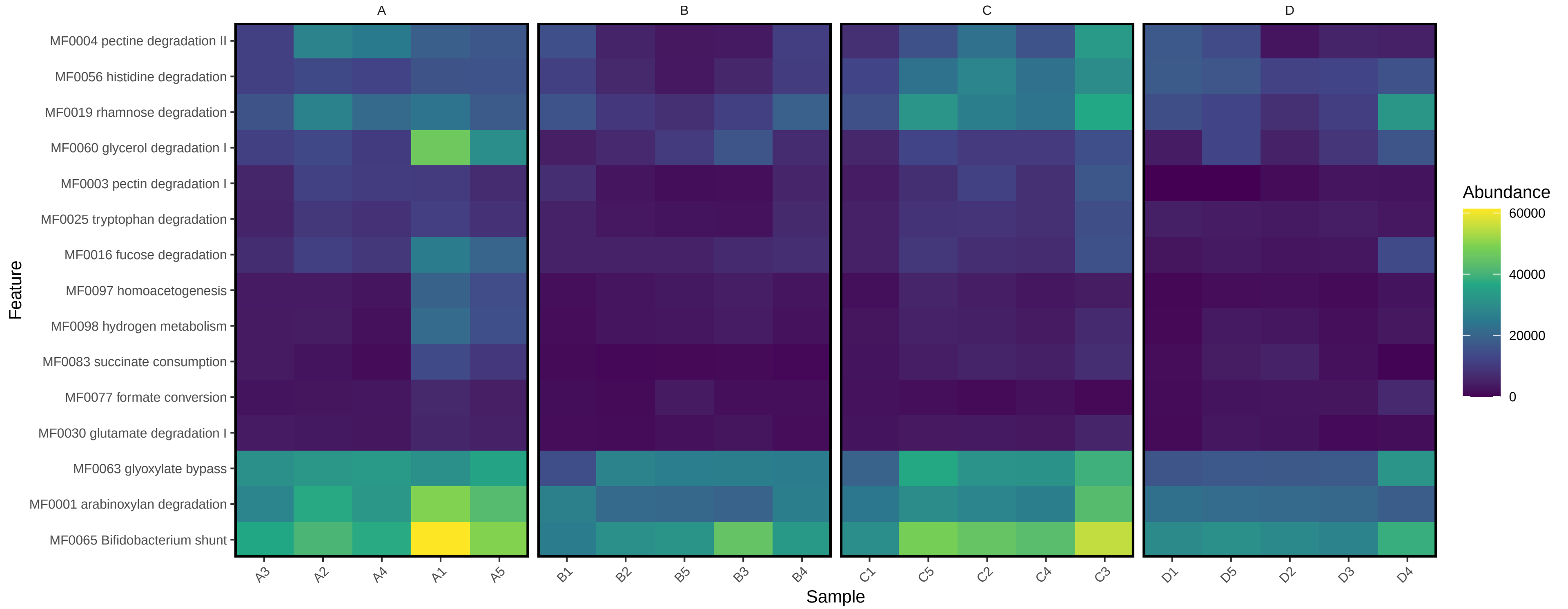

A
B
C
D
Feature
MF0004 pectine degradation II
MF0056 histidine degradation
MF0019 rhamnose degradation
MF0060 glycerol degradation I
MF0003 pectin degradation I
MF0025 tryptophan degradation
MF0016 fucose degradation
MF0097 homoacetogenesis
MF0098 hydrogen metabolism
MF0083 succinate consumption
MF0077 formate conversion
MF0030 glutamate degradation I
MF0063 glyoxylate bypass
MF0001 arabinoxylan degradation
MF0065 Bifidobacterium shunt
A3
A2
A4
A1
A5
B1
B2
B5
B3
B4
C1
C5
C2
C4
C3
D1
D5
D2
D3
D4
Sample
Abundance
60000
40000
20000
0
